# Synergistic Potential of Immune Checkpoint Inhibitor Combined with Neutrophil-Targeted Therapy in Cancer Immunotherapy

**DOI:** 10.34133/research.1210

**Published:** 2026-04-14

**Authors:** Huifang Shi, Jiaan Wang, Ziyi Wang, Yuanyuan Li, Tao Yu, Yan Qin, Jie Lv

**Affiliations:** ^1^ Clinical Laboratory, The Rizhao People’s Hospital Affiliated to Jining Medical University, Rizhao 276826, Shandong, China.; ^2^ Blood Transfusion Department, The Rizhao People’s Hospital Affiliated to Jining Medical University, Rizhao 276826, Shandong, China.; ^3^ Department of Anesthesiology, The Rizhao People’s Hospital Affiliated to Jining Medical University, Rizhao 276826, Shandong, China.; ^4^ Center for Vascular Biology and Translational Medicine, Institute for Translational Medicine, The Affiliated Hospital of Qingdao University, Qingdao 266021, China.

## Abstract

Recently, immune checkpoint inhibitors (ICIs) have demonstrated substantial benefits and potential by enhancing antitumor immune responses via the normalization of host T cell activity. Nonetheless, a considerable proportion of patients were tolerant to ICI therapy, thereby restricting its broader application in cancer treatment. Identifying the key barriers responsible for resistance to ICI therapy has led to the development of combination therapies with ICIs as a promising approach to overcome these challenges, providing new directions for ICI-based cancer immunotherapy. Neutrophils within the tumor microenvironment are considered a critical factor contributing to poor responses to ICI treatment, and combining ICIs with neutrophil-targeted therapy has shown remarkable efficacy and promise. Consequently, this review discusses the diverse roles of neutrophils in cancer immunotherapy, outlines the synergistic mechanisms of targeting neutrophils in conjunction with ICIs, highlights relevant clinical trials of this combination strategy, and discusses current limitations and future directions. Overall, this review aims to provide new insights into effective strategies for ICI therapy.

## Introduction

Cancer immunotherapy harnesses the host immunity system to effectively target and eliminate cancer cells, representing a revolutionary advancement in oncology [1]. The advent of immune checkpoint inhibitors (ICIs) has brought unprecedented progress to the field, with ICIs now incorporated into first-line treatments for various solid tumors and hematologic malignancies [2–4]. Several ICIs have received approval from the US Food and Drug Administration (FDA). Ipilimumab, the first authorized ICI for treating melanoma by FDA in 2011, targets cytotoxic T lymphocyte-associated protein 4 (CTLA-4) for the treatment of melanoma [5]. Subsequently, the anti-programmed cell death 1 (PD-1) antibodies, pembrolizumab and nivoluma, were granted for a variety of solid tumors and hematologic cancers. Additionally, since 2016, the anti-programmed death-ligand 1 (PD-L1) antibodies atezolizumab, durvalumab, and avelumab have been authorized [6]. Therapies targeting PD-1 and PD-L1 have generally demonstrated superior clinical efficacy and better tolerability compared to anti-CTLA-4 agents, with studies showing that anti-PD-1 monotherapy obviously prolonged survival in patients with non-small cell lung cancer (NSCLC) [7]. Despite the substantial survival benefits provided by ICIs across multiple cancer types, many patients were either unresponsive or failure to sustain a durable response [8]. This tolerance to ICIs is perhaps due to tumor-intrinsic factors, such as tumor mutational burden (TMB) and defective signaling pathways, as well as tumor-extrinsic factors including the tumor microenvironment, human microbiome, and metabolic status [9–11]. Consequently, such resistance limits the broader clinical application of ICIs. Understanding the underlying mechanism of resistance could help distinguish responders from nonresponders and inspire strategies to convert nonresponders into responders.

Lymphocytes, such as T cells and natural killer cells (NK cells), form the foundation of ICI efficacy, not only correlating with favorable outcomes but also being crucial for effective immunotherapy [12–15]. Besides, myeloid cells—particularly neutrophils—have recently been identified as essential players in cancer progression and resistance to ICIs [16]. Tumor-infiltrating neutrophils (TINs) are strongly associated with resistance to ICI therapy, whereas low levels of polymorphonuclear myeloid-derived suppressor cells (PMN-MDSCs) predict a favorable response [17,18]. Neutrophils are abundant in many cancers and can adopt diverse functional phenotypes that either promote or inhibit tumor growth [19]. To address these challenges, researchers are exploring strategies to reprogram neutrophils toward an antitumor state or to combine ICIs with other neutrophil-modulating approaches. Such combined regimens aim to enhance antitumor immunity, overcome PD-1/PD-L1 resistance, and broaden the population of patients who benefit from immunotherapy.

This review provides a comprehensive overview of the role of neutrophils in cancer immunotherapy, highlighting the synergy mechanisms underlying the combination of neutrophil-targeted therapies and ICIs. Moreover, ongoing and emerging clinical trials investigating these combination therapies are summarized and discussed. A deeper understanding of neutrophils and their integration into combination therapies holds substantial promise for advancing cancer immunotherapy.

## Functional Diversity of Neutrophils in Cancer Immunotherapy

### An overview of neutrophils in cancer

Traditionally, neutrophils are recognized as short-lived defenders against infections in the innate immune system. However, this view has been revised by findings indicating that neutrophils have a much longer lifespan [20] and exhibit more complex functions and phenotypes, as revealed through single-cell RNA sequencing (scRNA-seq) and transcriptome analyses [21,22]. Moreover, under pathological conditions such as cancer, the complexity and plasticity of neutrophils were further enhanced. Tumor-associated neutrophils (TANs) were previously believed to perform tumor-promoting roles, given the positive association between high TANs infiltration and poor prognosis [23]. Advances in understanding TANs have benefited from the identification of multiple transcriptional subtypes and their capability to polarize into diverse phenotypes. scRNA-seq analyses of human and mouse lung cancer have shown that TANs display a continuous phenotypic spectrum, comprising 5 subpopulations in humans and 6 in mice. Furthermore, neutrophil subtypes across species conserved gene expression patterns: canonical neutrophil markers (such as MMP9 and S100A8/9) are largely restricted to h/mN1 neutrophils, while h/mN2 neutrophils share a common type I interferon (IFN) response gene signature [24]. A single-cell transcriptomic study of neutrophils from 225 samples across 17 cancer types identified 10 distinct neutrophil states—including HLA-DR^+^CD74^+^, S100A12^+^, TXNIP^+^, CXCR2^+^, VEGFA^+^SPP1^+^, CXCL8^+^IL1B^+^, MMP9^+^, NFKBIZ^+^HIF1A^+^, IFIT1^+^ISG15^+^, and ARG1^+^ neutrophils—that are implicated in angiogenesis, inflammation, and antigen presentation [25]. Neutrophil subtypes vary dynamically across tumor types and conditions, reflecting their capacity for transcriptional reprogramming. Although a consensus nomenclature for TAN states is still lacking, it is widely accepted that TANs can exert both protumor and antitumor effects. Fridlender et al. [26] introduced the antitumor neutrophils (N1)/protumor neutrophils (N2) terminology to indicate a polarization state akin to the M1/M2 classification of macrophages. N1 neutrophils in tumors are classified as neutrophils to possess activities such as tumor-cytotoxic activity, metastasis suppression, and immunostimulation, whereas N2 neutrophils are associated with tumor promotion, immunosuppression, and metastasis [27]. Beyond functional distinction, N1 and N2 neutrophils also display distinct phenotypic profiles (Table 1).

**Table 1.** Basic features of N1 and N2 neutrophils in human

Characteristic	N1	N2
Surface markers		
CD14	−	−
CD15	+	+
CD66b	+	+
CD16	+	+
CD11b	High	Intermediate
SiglecF	Low	High
Physical properties		
Density gradient	High density	Low density
Nuclear morphology	Segmented	Banded, ringed, or segmented
FSC	Low	High
Effectors or enzymes		
Myeloperoxidase (MPO)	Low	High
Arginase (Arg)	Low	High
Reactive oxygen species (ROS)	Low	High
Suspected role in cancer	Antitumor	Protumor

As early as the 1980s, elevated neutrophil levels were linked to poor prognosis, increased metastasis risk, and reduced survival in nonhematological cancers [28,29]. More recently, the neutrophil-to-lymphocyte ratio (NLR) has emerged as an important prognostic and predictive indicator in patients with NSCLC [30], rectal cancer [31], thyroid cancer [32], and breast cancer [33], although its routine clinical application remains limited [34]. Nonetheless, the prognostic value and functional roles of neutrophils vary across various cancer types. For instance, high neutrophil infiltration is an adverse prognostic indicator in gastric cancer and metastatic renal cell carcinoma (mRCC) treated with targeted therapy [35,36], whereas increased neutrophil infiltration tends to correlate with better overall survival in gastric cancer patients undergoing postoperative chemotherapy [37]. Intriguingly, meta-analyses have shown inconsistent prognostic values for TANs in colorectal cancer (CRC) [38,39]. Such inconsistency may be attributed to the spatiotemporal dynamics of neutrophils within the tumor microenvironment during cancer progression, as well as the presence of distinct TAN subsets. For example, Peng et al. found that higher infiltration of intrastromal neutrophils in early-stage lung cancer predicts better prognosis and disease-free survival, while elevated intratumoral neutrophils in advanced stages are associated with poorer outcomes [40]. Similarly, various neutrophil subpopulations have been identified at different stages of CRC, showing phenotypic shifts in response to signals derived from tumors and showing variable associations with patient survival [41]. In brief, neutrophils in the context of immunotherapy can exhibit either antitumor or protumor functions (Table 2).

**Table 2.** Overview of neutrophil functions in cancer immunotherapy

Cancer type	Model	Mechanism	Reference
Antitumor roles			
Lung adenocarcinoma	KP lung tumor; MC38 tumor-bearing mice	Anti-PD-1 or anti-CD40 treatment expands a distinct neutrophil state with an Irf1 signature, and exert antitumor function dependent on DCs, IL-12, and IFNγ	[42]
CRC	CT-26 (MSS) tumor-bearing mice; neutrophils from CRC MSI-H patients	IFN**γ** treatment induces NET formation in neutrophils, which dampen tumor development and synergistically enhance the efficiency of anti-PD-1 therapy	[44]
Melanoma	B16F10 tumor-bearing mice; samples from melanoma patients	Type I IFN polarizes TAN into antitumor N1 phenotype with high expression of NETs, ICAMs, and TNFα	[45]
Melanoma	B16F10 tumor-bearing mice	IFNβ treatment inhibits the expression of CXCR4, MMP9, and VEGF in neutrophils, and thus delay tumor growth and angiogenesis	[46]
Mammary carcinoma; lung carcinoma	Mice 4T1 mammary carcinoma model; mice LLC lung carcinoma model	Type I IFN shifts neutrophil to an antimetastatic phenotype, and thus control the formation of lung metastases	[47]
Sarcoma	Mice 3-methylcholantrene-induced carcinogenesis; samples from human undifferentiated pleomorphic sarcoma	Neutrophils polarize UTC_**α**β_ and mediate type 1 antitumor immunity, and thus protecting against sarcomagenesis via IL-12/IFNγ pathway	[48]
Human 17 cancer types; murine 7 cancer types		Leucine treatment induces neutrophil into an antigen-presenting program, and thus enhances anti-PD-1 therapy	[25]
NSCLC	Samples from NSCLC patients; mouse NSCLC model	Neutrophil produces a high level of IL-8, which promotes the differentiation of CD74^high^SiglecF^low^ neutrophil, then boosts antitumor T cell activation through antigen-cross-presentation and enhances the efficacy of anti-PD-1 therapy	[50]
Melanoma	B16F10 tumor-bearing mice; B78H1 tumor-bearing mice; samples from patients that received immunotherapy either ipilimumab alone, nivolumab alone or both	Neutrophil eradicates antigen loss tumor variants through an iNOS-dependent mechanism in the context of T cell immunomodulation	[51]
Lung cancer	Samples from surgically resected lung cancer patients	TANs cross-talk with activated T cells to stimulate T cell proliferation and IFNγ release in the earliest stages of lung cancer	[52]
Bladder cancer	MB49 tumor-bearing mice	BCG treatment-induced NETs promote the recruitment of T cells and monocytes-macrophages, and thus prevent tumor growth	[74]
Protumor roles
NSCLC	Samples from patients with treatment-naived advanced NSCLC receiving ICI monotherapy	Elevated circulating LDNs with HGF/c-MET pathway activation mediate the resistance of ICI therapy	[60]
HCC	Samples from patients with HCC receiving anti-PD-1 immunotherapy; diethylnitrosamine (DEN)-induced rat HCC model	CD10^+^ALPL^+^ neutrophils induce T cells to an apparent irreversible exhaustion state, contributing to tumor immune escape from durable anti-PD-1 treatment	[62]
Bladder cancer	MB49 tumor-bearing mice	Neutrophils exert an immunosuppressive role on T cells in bladder cancer immune microenvironment and promote immune evasion of bladder cancer	[65]
Urothelial bladder carcinoma	Meta-analysis on the predicative value of NLR in ICI response and TANs roles; MB49 tumor-bearing mice	PGE2 synthesized by COX-2 in neutrophils up-regulates IDO1 in cancer cells and declines the efficacy of ICI treatment	[66]
HGSOC	Autochthonous HGSOC mouse model	Intratumoral neutrophils overactivated ER stress sensor IRE1α to facilitate early adaptive immune escape and resistance to PD-1 blockade	[67]
Melanoma; colon cancer; lung cancer; lymphoma	B16F10 tumor-bearing mice; MC38 tumor-bearing mice; EL4 tumor-bearing mice; LLC tumor-bearing mice; human melanoma tissues	CD300ld is essential for PMN-MDSCs to suppress T cells, and synergistical blockade of PD-1 and CD300ld enhance antitumor efficacy	[68]
Colorectal adenocarcinoma	MC38 tumor-bearing mice	NETs inhibit T cell responses through metabolic and functional exhaustion, and combined blockade of PD-L1 and NETs diminish tumor growth	[75]

PD-1, programmed cell death 1; IL-12, interleukin-12; IFNγ, interferon γ; MSS, microsatellite stable; CRC, colorectal cancer; NET, neutrophil extracellular trap; TAN, tumor-associated neutrophil; MMP9, matrix metalloproteinase 9; VEGF, vascular endothelial growth factor; NSCLC, non-small cell lung cancer; iNOS, inducible nitric oxide synthase; BCG, Bacillus Calmette–Guerin; ICI, immune checkpoint inhibitor; LDN, low-density neutrophil; NLR, neutrophil-to-lymphocyte ratio; PGE2, prostaglandin; COX-2, cyclooxygenase-2; HGSOC, high-grade serous ovarian cancer; ER, endoplasmic reticulum; MDSC, myeloid-derived suppressor cell

### Antitumor capabilities of neutrophils in cancer immunotherapy

**Fig. 1.**
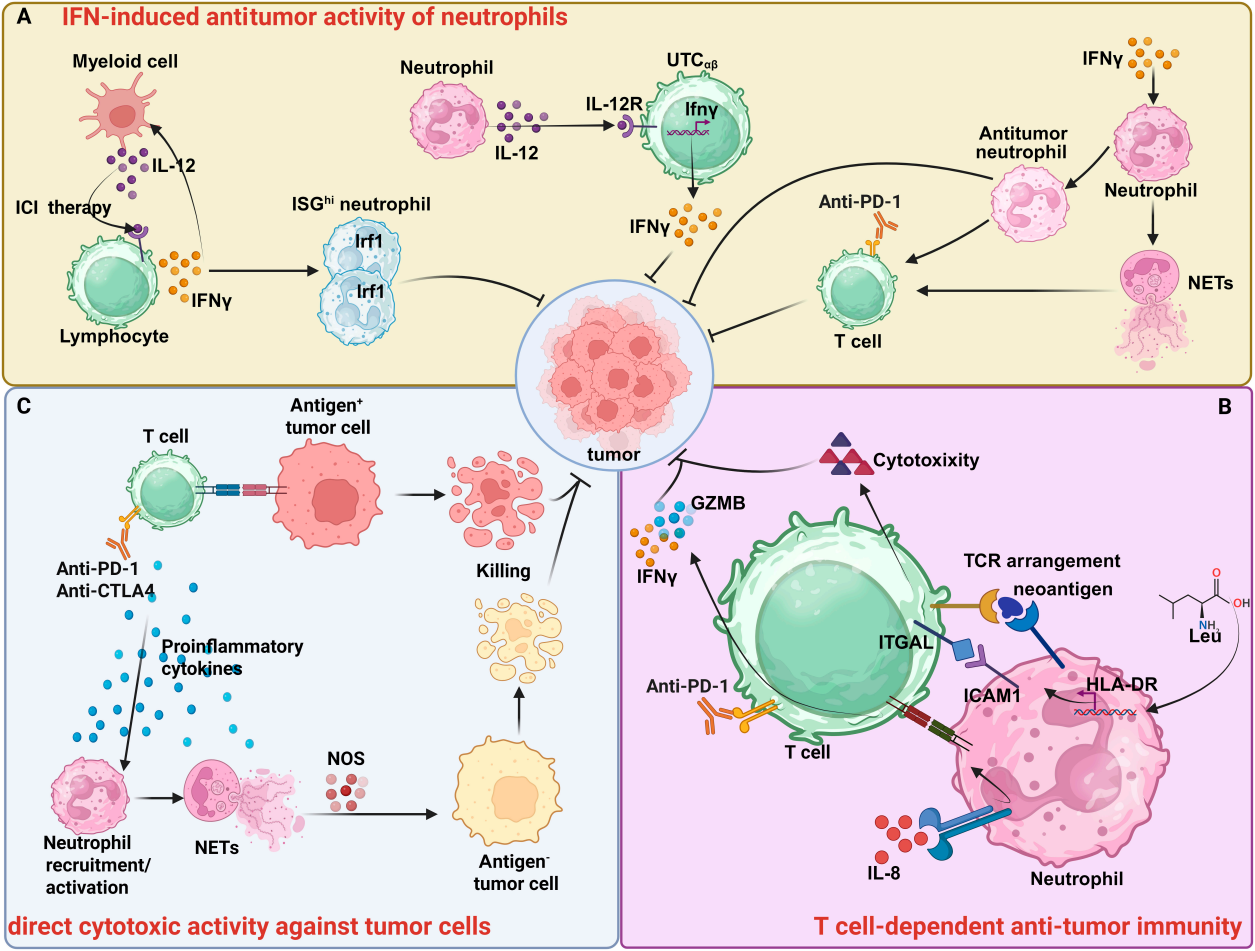
Antitumor capabilities of neutrophils in cancer immunotherapy. Neutrophils are implicated in various antitumor mechanisms, including interferon (IFN)-induced antitumor responses, direct cytotoxic effects, and T-cell-mediated antitumor immunity. (A) Following successful immune checkpoint inhibitor (ICI) therapy, neutrophils exhibiting an IFN-stimulated gene (ISG) signature proliferate to suppress tumor growth. The IL-12/IFNγ axis facilitates the polarization of CD4^−^CD8^−^ unconventional αβ T cells (UTC_αβ_) by neutrophils, thereby promoting antitumor immunity. IFNγ administration augments neutrophil antitumor activity either directly or via the induction of neutrophil extracellular traps (NETs). (B) Neutrophil-derived interleukin-8 (IL-8) or leucine exposure enhances the antigen-presenting capacity of neutrophils, thereby boosting T cell activation against tumors. (C) In the context of T cell-based immunotherapies, neutrophils eradicate antigen-negative tumor cells through an inducible nitric oxide synthase (iNOS)-dependent pathway. (Created with BioRender.com.)

Successful cancer immunotherapy has been linked to an increase in neutrophil levels [42]. Accumulating evidence indicates that neutrophils can directly eliminate tumor cells and enhance antitumor immunity (Fig. 1). The IFN signaling pathway, which includes type I IFNs (IFNα and IFNβ) and type II IFN (IFNγ), plays a central role in regulating tumor immune responses [43]. IFNs are widely acknowledged for their crucial functions in tumor immune surveillance, directly and indirectly inhibiting tumor progression via functioning on both tumor cells and immune cells (Fig. 1A). For example, PD-1 inhibition or anti-CD40 treatment in MC38 tumors increased the frequency of a specific Sell^hi^ neutrophil subset exhibiting an IFN-stimulated gene signature, thereby controlling tumor growth [42]. Administration of IFNγ strengthened the anti-PD-1-induced antitumor cytotoxic effect, accompanied by increased formation of neutrophil extracellular traps (NETs) and elevated apoptosis in microsatellite stable (MSS) CRC cell lines [44]. Another mechanism through which IFNs modify neutrophils involves polarizing TANs toward an antitumor property in both murine models and melanoma patients [45]. Additionally, in response to endogenous IFNs, neutrophils can inhibit tumor angiogenesis and metastasis by suppressing genes encoding proangiogenic and prometastatic factors such as vascular endothelial growth factor (VEGF) and MMP9 [46,47]. In a mouse model of 3-MCA-induced sarcoma, neutrophils were shown to polarize CD4^−^CD8^−^ unconventional αβ T cells (UTC_αβ_) and mediate type 1 antitumor immunity, thereby protecting against sarcomagenesis via the IL-12/IFNγ pathway [48]. Overall, IFNs induce functional and phenotypic transformations in neutrophils that contribute to the inhibition of tumor progression. Additionally, IFN therapy has been reported to disrupt neutrophil recruitment via limiting the expression of CXCR2 and CXCR4 [46,49].

Beyond IFN-mediated mechanisms, the antitumor capability of neutrophils that depends on T cells is also considered an important pathway for inducing tumor cell killing (Fig. 1B). For instance, improved survival and better responses to immunotherapy have been positively correlated with HLA-DR^+^ neutrophils exhibiting antigen-presenting capabilities across 8 immunotherapy cohorts, including patients with skin cutaneous melanoma, hepatocellular carcinoma (HCC), stomach adenocarcinoma, bladder cancer, and NSCLC [25]. Mechanistically, these HLA-DR^+^ neutrophils colocalize with effector memory T cells and central memory T cells that exert antitumor T cell activity. Leucine administration was shown to enhance the antigen-presenting abilities of neutrophils through metabolism-epigenetic regulation and mitochondrial remodeling [25]. Similarly, IL-8 produced by neutrophils prompts the differentiation of CD74^high^ neutrophils, which boost the response of T cell against tumors through antigen cross-presentation and are associated with favorable outcomes following ICI therapy in NSCLC [50]. In addition to antigen presentation, neutrophils have recently been found to eradicate antigen-negative tumor cells through an inducible nitric oxide synthase (iNOS)-dependent mechanism during T-cell-based immunotherapy (Fig. 1C) [51]. Another study demonstrated that neutrophils frequently colocalize with T cells and APCs, and TANs at the early stage of lung cancer exhibit strong phagocytic activity and stimulate antigen-nonspecific T cell proliferation [52].

Considering the various subtypes and functions of neutrophils in cancer, strategies have been developed to harness or reprogram them to eliminate tumors and reduce metastasis. For example, exposure to β-glucan, a prototypical agonist of trained immunity derived from fungus, endowed neutrophils with an antitumor phenotype through epigenetic and transcriptomic changes during granulopoiesis [53]. MnO_2_ nanoparticles can reprogram neutrophils to an N1 phenotype via the stimulator of interferon genes (STING) pathway, and when combined with *Salmonella*-mediated tumor immunotherapy, they substantially enhance anti-neoplastic efficacy, completely inhibiting tumor progression within 20 d and improving survival rates over 40 d [54]. A neutrophil-activating therapy consisting of an anti-CD40 agonist, tumor necrosis factor (TNF), and a tumor-targeting antibody has been shown to promote the clearance of various types of tumors by activating the alternative complement pathway, sensitizing C5a-C5AR1 signaling to produce leukotriene B4, which in turn induces the generation of reactive oxygen species (ROS) [55]. Leveraging the antitumor properties of neutrophils and their capability to traverse the blood–brain–tumor barrier, a CAR-neutrophil biomimetic nanotherapeutic delivery platform has been engineered to enable noninvasive, targeted delivery of nanoscale pharmaceuticals to glioblastoma, while minimizing proinflammatory responses at the delivery loci [56].

### Protumor capabilities of neutrophils in cancer immunotherapy

As noted above, neutrophil infiltration is prevalent in solid neoplasms and is often correlated with increased tumor burden and poorer overall survival [57,58]. Moreover, the limited efficacy of ICIs has been closely associated with neutrophil activity [59]. An elevated number of low-density neutrophils (LDNs) is indicative of primary resistance to ICI monotherapy in individuals with NSCLC [60].

**Fig. 2.**
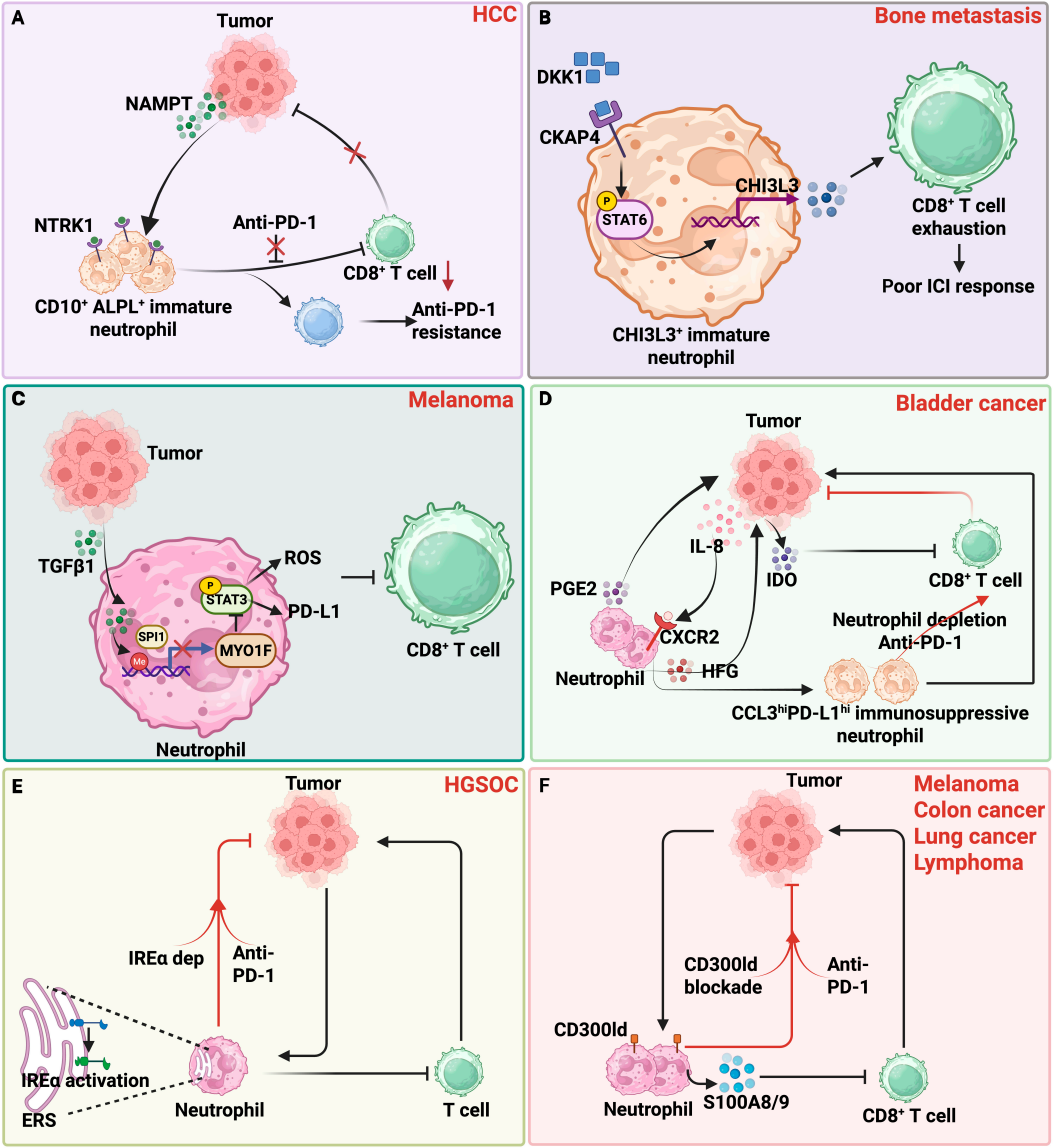
Mechanisms of neutrophil-mediated immunosuppression in tumor immune resistance against immunotherapy. (A) Tumor-derived factors such as NAMPT and IL-8 reprogram neutrophils to induce irreversible T cell exhaustion or reduce T cell activation in hepatocellular carcinoma (HCC). (B) During bone metastasis, dickkopf1 (DKK1) induced a transition of neutrophils into an immature and immunosuppressive state through the CKAP4–STAT6 pathway, which increased CHI3L3 secretion by neutrophils, and thus suppressed the antitumor response of CD8^+^ T cells. (C) Tumor-derived TGFβ1 reduced MYO1F expression by disrupting the interaction between SPI1 and Myo1f through DNA methylation, leading to increased PD-L1 and ROS expression in neutrophils dependent on the STAT3 signaling pathway. (D) In bladder cancer, cancer cell-secreted IL-8 induced the formation of a super-immunosuppressive CCL3^high^PD-L1^high^ subset, and a synergistic antitumor effect was achieved by neutrophil elimination in combination with anti-PD-1 antibody, and prostaglandin E2 (PGE2) secreted by tumor-associated neutrophils (TANs) impairs T cell function through IDO1 expression in cancer cells. (E) The endoplasmic reticulum (ER) stress sensor IRE1α is hyperactivated in neutrophils, rendering them insensitive to PD-1 blockade in high-grade serious ovarian cancer (HGSOC). (F) Under various tumor conditions, the expression of CD300ld in neutrophils is up-regulated, promoting the S100A8/A9 signaling pathway, thereby inhibiting the CD8^+^ T cell immune response. (Created with BioRender.com.)

The immunosuppressive properties of neutrophils are subtly hijacked by tumors to facilitate resistance against immunotherapy (Fig. 2). A key mechanism by which tumors evade immune surveillance is the induction of T cell exhaustion, particularly in CD8^+^ T cells. While PD-1 blockade can rejuvenate exhausted CD8^+^ T cells and improve therapeutic outcome [61], tumors can subvert this process. In HCC, for instance, tumors reprogram neutrophils into an immature CD10^+^ALPL^+^ phenotype via the nicotinamide phosphoribosyltransferase (NAMPT)/NTRK1 pathways, causing irreversible T cell anergy and facilitating tumors evasion from sustained anti-PD-1 immunotherapy (Fig. 2A) [62]. Recently, Shi et al. [63] revealed that chitinase-3-like protein 3^+^ (CHI3L3^+^) immature neutrophils impair ICI therapy in bone metastases from various malignancies. This effect is mediated by dickkopf1 (DKK1), which drives neutrophils into an immature, immunosuppressive state via the CKAP4–STAT6 pathway, thereby increasing CHI3L3 secretion and suppressing CD8^+^ T cell responses (Fig. 2B). Another investigation revealed that tumor-derived TGFβ1 diminished MYO1F expression by interfering with SPI1 binding of the Myo1f promoter through DNA methylation, resulting in neutrophils with STAT3-dependent immunosuppressive activity that contributes to CD8^+^ T cell exhaustion and limits ICI efficacy (Fig. 2C) [64]. Additionally, bladder cancer cell-derived IL-8 promotes the generation of highly immunosuppressive CCL3^high^PD-L1^high^ neutrophil subset, where combining neutrophil depletion with anti-PD-1 antibodies augments the proportion and cytolytic activity of CD8^+^ T cells, producing a synergistic antitumor effect (Fig. 2D) [65]. Consistent with this finding, high TAN infiltration correlates with poor ICI response in urothelial bladder carcinoma (UBC), and a deep mechanism exploration reveals that TAN-secreted prostaglandin E2 (PGE2) up-regulates IDO1 in cancer cells via the PKC–GSK pathway, impairing T cell function (Fig. 2D) [66]. In high-grade serous ovarian cancer, overactivation of the endoplasmic reticulum stress sensor IRE1α in neutrophils renders them insensitive to PD-1 blockade (Fig. 2E) [67]. CD300ld, a protein predominantly expressed in myeloid cells and highly abundant in neutrophils, is obviously up-regulated in the tumor microenvironment and modulates neutrophil activity through the STAT3–S100A8/A9 pathway (Fig. 2F). CD300ld knockout notably reduces genes associated with neutrophil migration and modulates ARG1 expression, thereby attenuating T cell activation [68]. Thus, neutrophil-mediated T cell immunosuppression represents a major obstacle to ICI efficacy. Furthermore, aberrant expression of immunoregulatory checkpoint proteins such as PD-L1 and V-domain Ig suppressor of T cell activation on neutrophil membranes may contribute to ICI resistance as well [69,70].

**Fig. 3.**
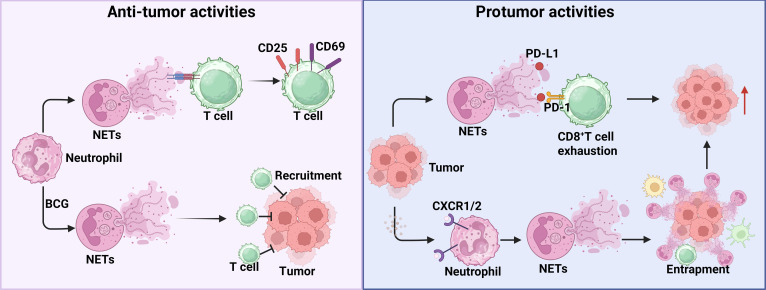
Dual roles of NETs in tumor immune response. The left panel illustrates the antitumor functions of NETs, where neutrophil-derived NETs interact with T cells, lowering their activation threshold by increasing the expression of CD25 and CD69. Moreover, Bacillus Calmette–Guerin (BCG) administration triggers NET formation, thereby effectively impeding tumor progression. The right panel depicts the protumor roles of NETs. Tumors manipulate NETs to induce CD8^+^ T cell exhaustion via the PD-L1/PD-1 pathway or to shield tumor cells from immune cytotoxicity. (Created with BioRender.com.)

When discussing the functions of neutrophils in cancer, NET formation is a critical biological phenomenon that warrants attention. NETs are intricate, filamentous extracellular structures generated by neutrophils, comprising DNA, histones, and cytotoxic enzymes. Their impact on tumor biology is context-dependent, varying with neutrophil subtype, cancer type, and disease stage, and NETs can exhibit both protumor and antitumor effects under different conditions. For example, lipopolysaccharide-induced NETs were shown to promote phagocytosis of CRC cells by down-regulating CD24 in a protease-activated receptor 2-dependent manner, thereby inhibiting CRC liver metastasis [71]. Conversely, NETs can facilitate lung metastasis in HCC. Mechanically, HCC cells reduce production of histidine-rich glycoprotein (HRG), which normally restrains IL-8-MAPK and NF-κB signaling and ROS generation; loss of HRG leads to dysregulated NET formation [72]. Similarly, NETs also exhibit dualistic functions in tumor immune responses (Fig. 3). Earlier studies highlighted the immune-stimulatory potential of NETs. For example, NETs released by human neutrophils attenuate the activation threshold of T cells to augment their immune response by up-regulating CD25 and CD69 as well as phosphorylating the TCR-associated signaling kinase ZAP70 [73]. Bacillus Calmette–Guerin therapy, a highly valuable treatment for bladder cancer, induces NET formation via IL-8 and TNFα and subsequently controls tumor progression by increasing the infiltration of immunocytes [74]. In contrast, the protumor effect of NETs in tumor immune response are increasingly elucidated. Kaltenmeier et al. [75] identified that ischemia/reperfusion (I/R)-induced NETs expressed PD-L1, which impairs T cell function and metabolism through PD-L1/PD-1 signaling, thereby dampening their response efficacy. Another proposed mechanism suggests that NETs can trap tumor cells and promote the dissemination of micrometastases to distant organs [76]. Consequently, the physical barrier formed by NETs restricts direct interaction between neoplastic cells and cytotoxic immune cells, diminishing the effectiveness of ICI therapies. Although substantial progress has been made in understanding the multifaceted roles of NETs in tumor immunity, it remains unclear which specific signals or factors determine whether NETs exert pro- or antitumor effects [77].

## Synergy of Targeting Neutrophils with ICIs

### Obstacles of ICI monotherapy in anticancer therapy

The application of ICIs has brought tremendous advances to the field of tumor treatment by effectively potentiating host immune effector cell against malignant tumors. ICIs predominantly employed in clinical practice target the CTLA-4 and PD-1/PD-L1 pathways, yielding obvious improvements over conventional cancer therapies, including extended survival and sustained remissions in some metastatic cancer patients. Despite these substantial advancements across various malignancies, the limitations of ICIs remain a critical consideration. A primary challenge is that only a subset of patients responds favorably to ICI therapy, as a vast number of cancer patients display primary or acquired resistance [78]. Additionally, it may introduce onset of immune-related adverse events (irAEs), ranging from asymptomatic or subclinical symptoms to severe, life-threatening conditions [79,80]. Consequently, ICIs encounter multiple hurdles that necessitate urgent resolution to broaden their therapeutic applicability and mitigate their immune-related toxicities.

The resistance mechanisms to ICIs can be classified into 3 primary categories: tumor-intrinsic factors, the tumor microenvironment, and host-extrinsic factors [9,78]. Defects in antigen presentation, loss of neoantigens, and aberrant activation of signaling pathways—such as IFN signaling, phosphatidylinositol-3-kinase (PI3K) signaling, and Wnt/β-catenin signaling pathway—represent tumor-intrinsic factors that limit ICI efficacy [78]. The tumor niche, encompassing stromal constituents, immune cells, metabolism, and microbiota, also plays a key role in modulating responses to ICIs. Of note, immune-related elements are pivotal to ICI resistance, as dynamic interactions between immunocytes and neoplastic cells dictate the immunological landscape of tumors and affect their susceptibility to ICIs. T cells are the principal participants of ICI therapy among numerous immune cells, and their presence and function at the tumor margin and within the tumor are critical determinants of therapeutic outcomes. However, despite the fact that T cells are widely regarded as central to ICI responses, the induction and recruitment of immune cells with immunosuppressive activity, along with the up-regulation of negative regulatory factors, contribute to T cell dysfunction and subsequent ICI insensitivity. Immune cells that possess immunosuppressive properties, including MDSCs, TANs, regulatory T cells (Tregs), and tumor-associated macrophages (TAMs), are exploited by tumors to evade immune attack and ICI treatment [81]. In particular, TANs are important contributors to ICI resistance, and targeting TANs can not only mitigate the negative regulatory effects of neutrophils on immune effector cells and reverse ICI resistance, but also enhance ICI efficacy by reprogramming neutrophils. Based on distinct neutrophil-related targets, these strategies can be broadly categorized into the following: (a) inhibition of neutrophil recruitment, (b) neutrophil depletion, (c) modulation of suppressive reprogramming, and (d) promotion of differentiation. Several clinical studies have confirmed the safety and preliminary efficacy of these strategies to some extent [82]. Consequently, combining TAN-targeted approaches with ICIs represents a promising therapeutic strategy with considerable potential.

### Synergy of targeting neutrophils recruitment combined with ICIs

**Fig. 4.**
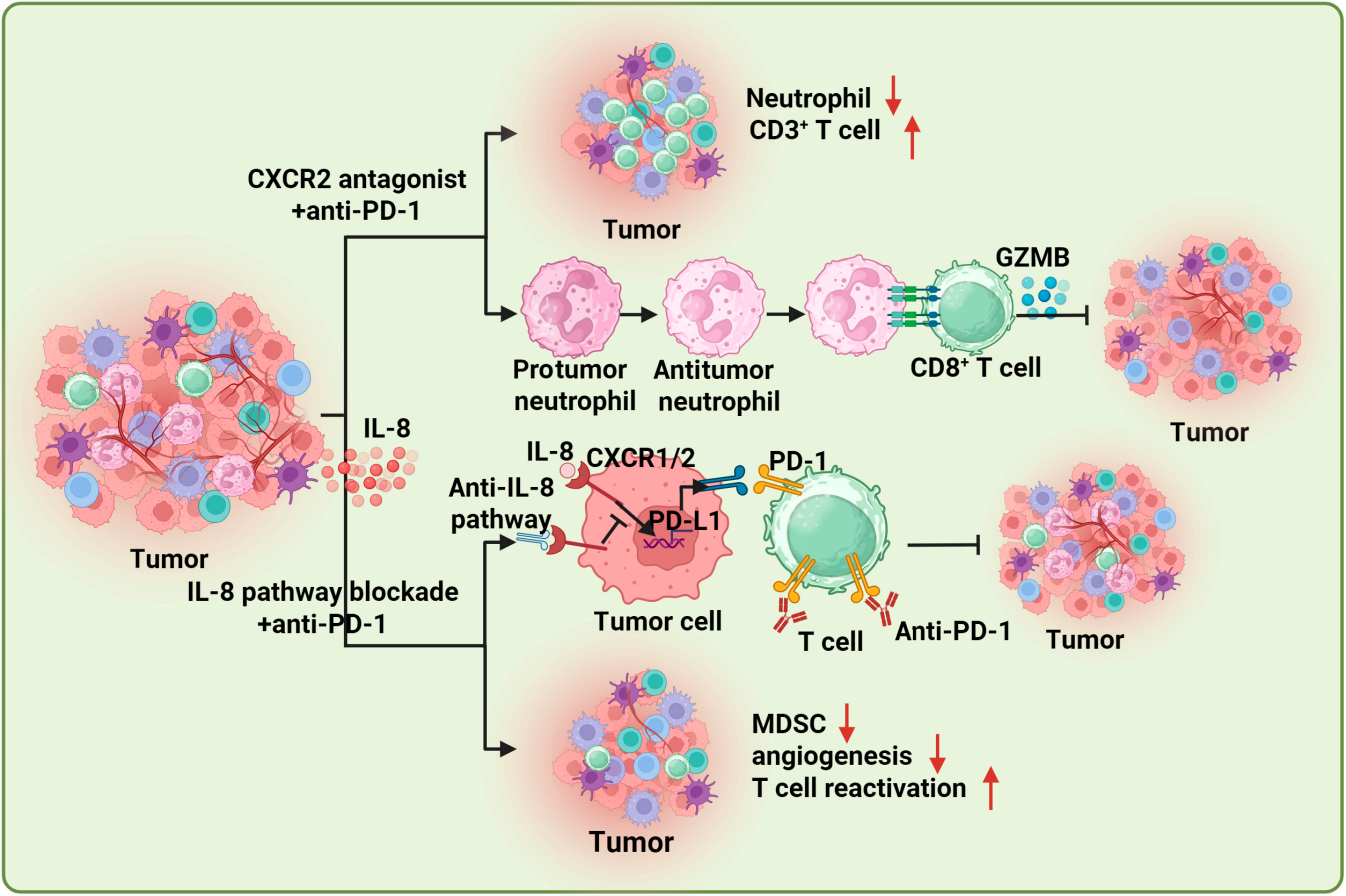
Synergistic effects of concurrently targeting IL-8/CXCR2 and ICIs. Firstly, the inhibition of CXCR2 in combination with anti-PD-1 therapy obviously enhances CD3^+^ T cell infiltration and increases the responsiveness to anti-PD-1 immunotherapy. This dual approach also reprograms neutrophils from a tumor-promoting to a tumor-inhibiting phenotype, thereby educating CD8^+^ T cells to enhance the efficacy of anti-PD-L1 blockade. Secondly, the blockade of the IL-8 pathway along with anti-PD-1 treatment reduces PD-L1 expression on neutrophils and tumor cells and synergistically amplifies the antitumor immune response of anti-PD-1 therapy. Furthermore, this combination strategy diminishes the recruitment of MDSCs, reduces angiogenesis, and promotes T cell reactivation. (Created with BioRender.com.)

The recruitment of neutrophils is primarily mediated by chemokine–receptor interactions. The most extensively studied receptors in this process are CXCR1 and CXCR2, which bind to ligands including CXCL1, CXCL2, CXCL3, CXCL5, CXCL6, CXCL7, and CXCL8 (also known as IL-8). Various strategies targeting CXCR1/2 or their ligands have been evaluated for cancer therapy, demonstrating synergistic effects in preclinical tumor models (Fig. 4). For example, Steele et al. [83] reported that CXCR2 signaling is up-regulated in myeloid cells and that simultaneous blockade of CXCR2 and PD-1 improved the efficacy of anti-PD-1 therapy. This suggests that inhibiting CXCR2 signaling may represent a promising therapeutic strategy to overcome ICI resistance and enhance immunotherapy outcomes. First, CXCR2 blockade reduces neutrophil infiltration—the primary immune function of CXCR2 in inflammatory neutrophil migration. Consequently, key protumor neutrophil activities are hindered, including angiogenesis (marked by elevated Bv8 and MMP9) and immunosuppression (reflected by increased ARG1, S100A9, and PD-L1) [84–87]. Additionally, CXCR2 inhibition enhances the infiltration and activation of XCR1^+^ cDC1 and CD3^+^ T cells in tumors, which resensitizes tumors to PD-1/PD-L1 blockade [83,88]. Interestingly, inconsistent with impaired neutrophil recruitment observed in a considerable amount of findings, the therapeutic benefit from concurrent inhibition of PD-1 and CXCR2 in nonalcoholic steatohepatitis–hepatocellular carcinoma (NASH–HCC) was accompanied by an elevation in the number of TANs and a phenotypic shift of TANs from tumor-promoting to tumor-suppressing states, but there is no evidence of similar systemic effects on circulating neutrophils [88]. However, the exact mechanism of selective reprogramming of TANs in tumors is still elusive. Additionally, the CT10 regulator of kinase-like (CRKL) gene was identified as a key mediator to mobilize TANs through the CRKL/β-catenin/VEGFα and CXCL1 pathways; blocking CRKL or its downstream effectors restored anti-PD-1 efficacy in murine tumor models [89]. Overall, these lines of evidence highlight the strong potential for synergistic targeting of neutrophil recruitment and ICIs for cancer patients that exhibit immunotherapy resistance.

**Fig. 5.**
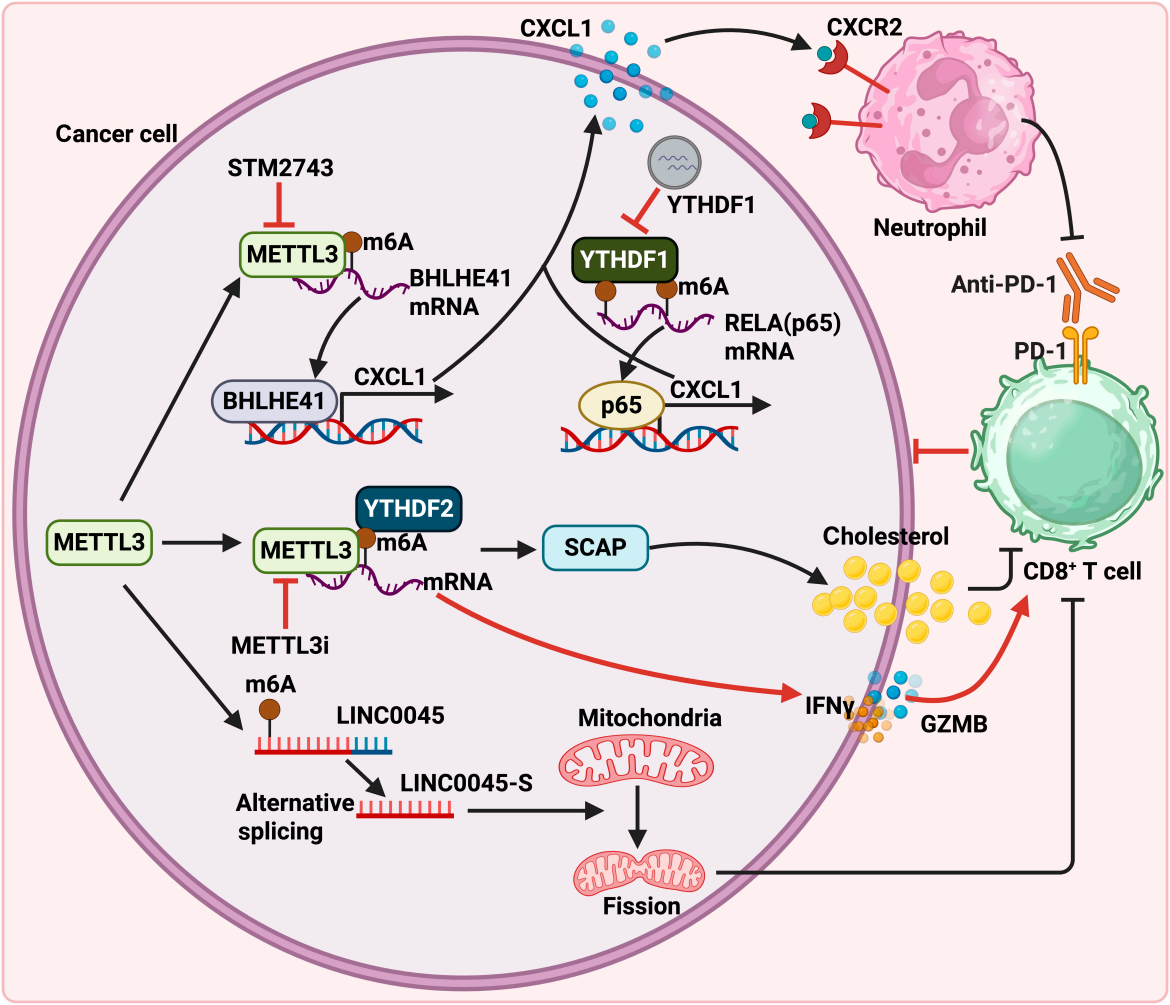
Synergistic mechanism of METTL3 or YTHDF1 inhibition in conjunction with anti-PD-1 therapy. Inhibiting METTL3 or YTHDF1 amplifies the efficacy of anti-PD-1 treatment by recruiting neutrophils through the METTL3–m6A–CXCL1 axis. Additionally, METTL3 knockdown increases the expression of IFNγ and GzmB, thereby enhancing the effectiveness of anti-PD-1 therapy via the m6A–YTHDF2 pathway. In addition, METTL3-mediated LINC0045 promotes the fission of mitochondria and therefore inhibits the activation of T cells. (Created with BioRender.com.)

Methyltransferase-like 3 (METTL3) is a pivotal enzyme responsible for N6-methyladenosine (m6A) modification, which plays a pivotal function in the advancement of tumorigenesis [90]. Of note, its immunoregulatory function in tumor immunology is also nonnegligible. By promoting m6A-dependent BHLHE41 expression, METTL3 drives CXCL1 transcription, and targeting METTL3 enhances the efficacy of anti-PD-1 treatment by leveraging the METTL3–m6A–CXCL1 axis, consequently promoting MDSC migration in CRC (Fig. 5) [91]. Simultaneously, the m6A reader YTH N6-methyladenosine RNA binding protein 1 (YTHDF1) enhances the translation of p65, which up-regulates CXCL1 expression and thereby facilitates MDSC recruitment through the CXCL1–CXCR2 axis (Fig. 5) [92]. However, it remains unclear whether an association exists between the transcription factors BHLHE41 and p65 in regulating CXCL1 transcription. Furthermore, beyond enhancing antitumor immunity via the m6A–CXCL1/CXCR2 axis, METTL3 inhibition also reduces cholesterol secretion and up-regulates the expression of IFN-γ and granzyme B (GzmB), thereby improving T cell function (Fig. 5) [93,94]. Consequently, targeting the epigenetic regulator METTL3 in combination with ICIs exhibits potential as a therapeutic modality for nonalcoholic fatty liver disease, melanoma, and colorectal adenocarcinoma [93,94]. Elsewhere, Yan et al. [90] demonstrated that METTL3-mediated alternative splicing of LINC00475 accelerates the development of glioma through mitochondrial fission (Fig. 5). Mitochondrial dynamics (fusion and fission) are essential for mitochondrial function, and targeting mitochondria appears beneficial for restoring antitumor immunity—for example, by modulating M1 polarization or restoring T cell function [95,96]. Given these insights, it is plausible that targeting METTL3-mediated splicing may also improve ICI efficacy. However, further experimental validation and clinical trials are needed to confirm this hypothesis.

Despite substantial progress in inhibiting chemokines, their receptors, or downstream signaling, a critical challenge remains: the relevant chemokines and receptors are also expressed on other cell types—such as monocytes, macrophages, fibroblasts, and endothelial cells—which may lead to off-target effects. Therefore, the potential for unpredictable effects on other cells remains a major concern for such combination strategies.

### Synergy of attenuating neutrophil immunosuppression combined with ICIs

Owing to the irreparable limitations of chemokine signaling blockade, it is imperative to identify more precise targets to mitigate neutrophil-mediated resistance in ICI therapy. Neutrophil-induced immunosuppression is a major obstacle to ICI efficacy, and various strategies have been explored to attenuate this suppression and enhance synergy with immunotherapy. TANs express the inhibitory molecule ARG1, which depletes L-arginine and thereby suppresses the antitumor activity of T cells. Consequently, ARG1 blockade restores CD8^+^ T cell function and enhances the efficacy of ICIs in pancreatic ductal adenocarcinoma (PDAC) [97]. Beyond ARG1, other immunosuppressive factors—such as neutrophil-derived PGE2—also impair T cell function; inhibiting PGE2 synthesis with celecoxib has been shown to improve the efficacy of ICI [66]. Given the complexity of neutrophil-mediated immunosuppression, targeting crucial regulators of this process represents a promising therapeutic strategy. For example, the poly(ADP-ribose) polymerase inhibitors olaparib and talazoparib—authorized primarily for BRCA1/2 mutated breast and ovarian malignancies [98], as well as histone deacetylase inhibitor entinostat—were shown to modulate MDSCs’ suppressive function by inhibiting ARG1, iNOS, and cyclooxygenase-2 (COX-2) expression, thereby enhancing anti-PD-1 efficacy in various cancer types [99–101]. Recent studies have identified several cell surface proteins—including CD200R, CD300ld, and the lectin-type oxidized LDL receptor 1 (LOX-1)—as key mediators of the immunosuppressive activity of PMN-MDSCs. Blocking CD200 signaling amplified anti-PD-1 efficacy by modulating MDSCs’ proliferation and suppression through activating STAT3 in PDAC [102]. The concurrent inhibition of CD300ld and PD-1 synergistically suppresses tumor growth by reversing the immunosuppressive tumor microenvironment. This effect is attributed to the essential role of up-regulated CD300ld in mediating the migration and immunosuppressive function of PMN-MDSCs via the STAT3–S100A8/A9 axis in tumor-bearing hosts [68]. Similarly, LOX-1 has been identified as a key regulator responsible for the immunosuppressive activity of PMN-MDSCs, with LOX-1^+^ cells exhibiting higher expression of DCFDA, ARG1, and iNOS compared to LOX-1^−^ cells [103,104]. Thus, targeting LOX-1 represents a promising strategy to attenuate neutrophil-mediated immunosuppression.

Besides, neutrophils in tumor microenvironment can also drive immunosuppression dependent on metabolic reprogramming. In particular, lipid metabolism is a key metabolic pathway that regulates neutrophil immunosuppressive function. Targeting fatty acid transport protein 2 (FATP2), a transporter facilitating arachidonic acid metabolism and endowing functions to PMN-MDSCs, or inhibiting arachidonic acid synthesis has emerged as promising targets [105,106]. Adeshakin et al. revealed that tumor cell-derived GM-CSF up-regulates FATP2 via STAT3 signaling, increasing lipid uptake and MDSC accumulation. This increased FATP2 conferred immunosuppressive activation of MDSCs via producing ROS, thus accelerating tumor progression. Therefore, FATP2 inhibition in MDSCs markedly reduces lipid accumulation and ROS generation, along with their immunosuppressive activity. Crucially, FATP2 blockade also sensitizes tumors to anti-PD-L1 therapy through increasing CD107a expression and decreasing PD-L1 on tumor-infiltrating CD8^+^ T cells [106]. Alternatively, another study discovered that FATP2 is exclusively up-regulated in PMN-MDSCs by GM-CSF through STAT5 (not STAT3) activation. Mechanistically, FATP2 enhances PMN-MDSC immunosuppression by promoting arachidonic acid uptake and PGE2 synthesis [107]. While both studies identify FATP2 as an essential modulator of PMN-MDSC immunosuppression, the reported upstream pathways differ, possibly due to tumor-type variations or distinct signaling contexts involving GM-CSF/STAT3/STAT5/FATP2 [106]. Regardless, both works highlight the therapeutic potential of combining FATP2 inhibition with ICIs. Beyond that, enhanced glycolysis has been shown to promote the immunosuppressive effects of neutrophils on effector T cells, thereby contributing to elevated tumor immune escape. STAT3 activation promotes anti-PD-1 resistance by driving hepatocyte serum amyloid A (SAA) secretion and neutrophil PD-L1 up-regulation via enhanced glycolysis, which collectively suppresses cytotoxic T cell function in a mouse HCC model [108]. Additionally, aconitate decarboxylase 1 (Acod1), a pivotal intermediate in the TCA cycle, is essential for blunting ferroptosis in TINs through the GM-CSF-JAK/STAT5-C/EBPβ axis, thereby facilitating lung metastasis of breast cancer; Acod1 ablation consequently amplifies the antimetastatic effect of ICI-reinvigorated cytotoxic T lymphocytes [109]. These work elucidate key mechanisms through which glucose metabolism contributes to immunotherapy resistance. Notably, dietary interventions are emerging as promising strategies to potentiate cancer therapy through metabolic modulation. For instance, leucine evokes neutrophils to an antigen-presenting program, which obviously improves anti-PD-1 treatment and stabilizes disease development in various murine cancer models [25]. Besides, the ketogenic diet has attracted increased interest for its potential in cancer treatment [110]. Synergistic effects have been observed when combining ICIs with supplementation of the ketone body β-hydroxybutyrate, the histone deacetylase inhibitor vorinostat, or a cyclic ketogenic diet, which reshape the tumor immune microenvironment to an antitumor state [111].

Due to the powerful role of NETs in immune suppression, therapeutic strategies targeting NETs have been developed. Considering the pivotal role of iron metabolism in NET formation, a transformable iron-chelating peptide–drug conjugate was designed to inhibit this process. Combined administration of this nanochelator with protein arginine deiminase 4 (PAD4) inhibitors amplifies the therapeutic benefit of anti-PD-L1 treatment [112]. Analogously, pharmacological inhibition of PAD4-mediated NETosis obviously increased tumor sensitivity to dual checkpoint inhibition involving PD-1 plus CTLA-4 [113]. CHI3L1, an immunosuppressive cytokine transcriptionally regulated by STAT3, has been identified as a direct inducer of neutrophil recruitment and NET formation. Targeting CHI3L1 improves response to ICIs in TNBC [114]. Remarkably, the application of DNase I to degrade NETs led to improved therapeutic efficiency of anti-PD-1 through fostering the infiltration and cytotoxicity of CD8^+^ T cells in a CRC model [115].

Notably, as demonstrated above, STAT3 plays a central role in PMN-MDSC function, highlighting the therapeutic potential of inhibiting the STAT3 pathway to overcome resistance to ICIs. Indeed, STAT3-targeted combination immunotherapy has shown encouraging results. For example, indirect STAT3 inhibition with dasatinib—which targets SRC/ABL—remarkably enhanced the efficacy of anti-CTLA-4 immunotherapy in head and neck squamous cell carcinoma (HNSCC) [116]. Furthermore, tumor-derived factors (including TNFα, IL-6, HMGB1, CCL20, and GM-CSF) and hypoxia up-regulate PD-L1 expression on neutrophils via the JAK/STAT3 pathway, thereby suppressing antitumor immunity [117]. Consequently, a JAK inhibitor (JAKi) reversed immune resistance and acted synergistically with dual ICI therapy (anti-PD-1 plus anti-CTLA-4) by remodeling the immunosuppressive tumor microenvironment [118]. It is also noteworthy that the TNF signaling pathway, as a key link between inflammation and cancer, critically regulates neutrophil activation and functional polarization. Specifically, TNFα up-regulates PD-L1 expression on neutrophils [119] and mediates the activation of neutrophils that promote gastric cancer progression via the TNFα/B7-H2/IL-17A axis by polarizing T-helper cell subsets [120]. Additionally, c-Rel, a member of the nuclear factor-κB (NF-κB) family, was recently identified as a myeloid checkpoint that controls the expression of protumor genes (such as ARG1, CEBPB, and iNOS) and combined inhibition of c-Rel and PD-1 exhibits superior efficacy compared to either treatment alone [121]. Mechanistic studies reveal that c-Rel can specify the MDSC signature gene expression dependent on c-Rel enhanceosome, representing an effective immunotherapeutic strategy for blocking immunosuppression of MDSCs.

Collectively, these preclinical findings highlight the therapeutic potential of suppressing neutrophil-mediated immunosuppression to enhance ICI efficacy.

### Synergy of facilitating neutrophils differentiation combined with ICIs

Due to their immature state, MDSCs are highly vulnerable to differentiation into mature phenotypes by agents such as all-trans retinoic acid (ATRA). ATRA, a derivative of vitamin A, is clinically employed and has obviously improved the prognosis of acute promyelocytic leukemia (APL) [122]. Besides, ATRA’s immunomodulatory properties have garnered considerable interest in certain solid tumors. Investigations demonstrate that ATRA notably reduces both the frequency and immunosuppression of MDSCs, thereby enhancing CD8^+^ T cell-mediated antitumor responses. Mechanistically, ATRA induces the synthesis of glutathione dependent on up-regulating glutathione synthase, resulting in ROS neutralization [123]. ROS not only directly participates in T cell inhibition by MDSCs, but also serves as one of the primary mediators preventing MDSCs from differentiating into mature state [124,125]. Therefore, the synergistic application of ICIs with ATRA was shown to augment immunotherapy efficacy in NSCLC and cervical cancer [126,127]. Similar to ATRA, vitamin D3 can also promote the differentiation of MDSCs toward a more mature phenotype and enhance T cell responses as well [128]. For example, treatment with 1α,25-dihydroxyvitamin D3 induces the differentiation of CD34^+^ progenitor cells—precursors of MDSCs—into dendritic cells (DCs) in patients with HNSCC [129]. In summary, these findings indicate that promoting neutrophil differentiation represents a promising therapeutic strategy to counteract neutrophil-mediated immune evasion in the context of ICI therapy.

### Synergy of neutrophil depletion combined with ICIs

Neutrophil depletion also exhibited impressive potential in enhancing the efficacy of ICIs. Studies indicate that chemotherapeutic agents can bring synergistic benefits for ICI therapies, though the underlying mechanisms remain ambiguous. Some research suggests that chemotherapy induces immunogenic cell death, thereby exposing tumor antigens and activating cytotoxic immune cells [130]. Others have shown that agents such as gemcitabine, cisplatin, and fluorouracil (5-FU) can concurrently deplete MDSCs and tumor cells, thereby improving therapeutic outcomes [131–133]. In particular, gemcitabine not only reduces MDSCs but also up-regulates PD-1 expression on T cells and PD-L1 expression on tumor cells, generating a synergistic antitumor effect when combined with PD-1/PD-L1 blockade [134]. Cisplatin-based chemotherapy can induce ferroptosis-mediated antitumor immunity by polarizing neutrophils toward an N1 phenotype, which enhances T cell recruitment and Th1 subset polarization to boost antitumor activity. This synergistic effect of cisplatin plus anti-PD-1 was abolished by the ferroptosis inhibitor ferrostatin-1 [135]. In addition, targeted antibodies designed to specifically deplete MDSCs have been developed to effectively eliminate neutrophils in tumor-bearing hosts. For instance, the anti-Gr-1 antibody is widely used to deplete MDSCs in murine models, while the humanized anti-CD33 antibody gemtuzumab ozogamicin induces MDSC-specific depletion in human cancers [136]. These strategies have been validated to mitigate the inhibitory impact of neutrophils on ICIs [136,137].

### Safety considerations of targeting neutrophils combined with ICIs

Currently, while several agents targeting neutrophils enhanced ICI efficiency, they also impaired neutrophil function or induced neutropenia as side effects. Although a low neutrophil-to-lymphocyte frequency is associated with improved overall survival and progression-free survival (PFS) in some cancer patients receiving ICIs, concerns persist regarding an increased risk of opportunistic infections, given the essential role of neutrophils in host immune defense. Moreover, indiscriminate interventions—such as neutrophil depletion or inhibition of neutrophil recruitment—that affect both protumor and antitumor neutrophil subsets may further elevate infection risk. For example, combining ICIs with chemotherapy has been associated with a higher incidence of irAEs, including pneumonia, pneumonitis, and upper respiratory tract infections [138]. Importantly, such treatment-related infections occur more frequently in real-world populations than reported in clinical trials, leading to worse clinical outcomes [139]. Therefore, further research is needed to improve the safety of combination therapies. A key priority is to precisely target protumor neutrophils without compromising host anti-infective defenses. Fortunately, the discovery of subcluster-specific neutrophil surface markers, such as CD300ld and LOX-1, bring promising therapeutic targets to specifically address the PMN-MDSC population. Among these therapeutic targets, CD300ld holds notable advantages. First, its expression is largely restricted to PMN-MDSCs. Second, CD300ld knockout suppresses the immunosuppressive activity of PMN-MDSCs on T cells without remarkably affecting normal development. Third, CD300ld is up-regulated across multiple human tumor types. Thus, targeting CD300ld exhibits high specificity, safety, and potential efficacy. Notably, antibody–drug conjugates (ADCs)—composed of a monoclonal antibody, a linker, and a cytotoxic payload—can enhance therapeutic efficacy while reducing systemic toxicity [140]. Given the selective expression of CD300ld on PMN-MDSCs and its elevated presence in tumor tissues, this transmembrane protein represents a promising target for ADC development. Theoretically, CD300ld-based ADCs could simultaneously deliver cytotoxic effects to PMN-MDSCs and tumor cells, thereby maximizing therapeutic benefit while minimizing off-target effects. Therefore, the development and evaluation of CD300ld-directed ADCs represent a valuable direction for future research.

Collectively, given the promising results from current studies targeting neutrophils in conjunction with ICIs, there is optimism that such combination strategies may enhance ICI efficacy in clinical practice. In addition, a neutrophil-targeted therapeutic approach is not confined to influencing a single neutrophil function but may potentially modulate multiple aspects of neutrophil activity. Nevertheless, majority of these studies remain at the preclinical stage, and rigorous evaluation of their clinical potential is essential in future research.

## Clinical Studies on Combining Neutrophil-Targeted Strategies with ICIs

Currently, numerous strategies aimed at targeting neutrophils are being explored for tumor immunotherapy, with several approaches undergoing clinical assessment in various cancer patients. The clinical trials registered on ClinicalTrials.gov are summarized in Table 3 (excluding terminated or withdrawn trials). These studies are being conducted across different phases and include patients with hematological malignancies and solid tumors. Additionally, some trials are evaluating the safety and efficacy of combining neutrophil-targeted agents with ICIs, chemotherapy, or other targeted therapies.

**Table 3.** Clinical trials concerning on the combined treatment of targeting both neutrophils and ICIs registered on clinicaltrials.gov

Mechanism	Target	Drug name	Combination therapy	Cancer	Phase	Enrollment (actual or estimated)	Last reported status	NCT number
Inhibiting recruitment	CXCR1/2	Navarixin	Pembrolizumab	Solid tumors Castration-resistant prostate cancer Microsatellite stable colorectal cancer Non-small cell lung cancer	II	107	Completed	NCT03473925
		SX-682	Nivolumab	Metastatic colon adenocarcinoma Metastatic colorectal carcinoma Metastatic rectal adenocarcinoma Stage III colon cancer AJCC v8 Stage III rectal cancer AJCC v8 Stage IIIA colon cancer AJCC v8 Stage IIIA rectal cancer AJCC v8 Stage IIIB colon cancer AJCC v8 Stage IIIB rectal cancer AJCC v8 Stage IIIC colon cancer AJCC v8 Stage IIIC rectal cancer AJCC v8 Stage IV colon cancer AJCC v8 Stage IV rectal cancer AJCC v8 Stage IVA colon cancer AJCC v8 Stage IVA rectal cancer AJCC v8 Stage IVB colon cancer AJCC v8 Stage IVB rectal cancer AJCC v8 Stage IVC colon cancer AJCC v8 Stage IVC rectal cancer AJCC v8 Unresectable colon adenocarcinoma Unresectable rectal adenocarcinoma	Ib/II	51	Recruiting	NCT04599140
		SX-682	Tislelizumab	Pancreatic cancer	II	25	Recruiting	NCT05604560
		SX-682	Nivolumab	Metastatic pancreatic ductal adenocarcinoma	I	20	Recruiting	NCT04477343
		SX-682	Pembrolizumab	Metastatic lung non-small cell carcinoma Stage IV lung cancer AJCC v8 Stage IIIC lung cancer AJCC v8 Recurrent lung non-small cell carcinoma	II	30	Recruiting	NCT05570825
		SX-682	Pembrolizumab	Melanoma stage III Melanoma stage IV	I	77	Recruiting	NCT03161431
		SX-682	Retifanlimab + TriAdeno vaccine + IL-15 agonist N-803	Metastatic colorectal cancer	I/II	60	Recruiting	NCT06149481
	IL-8	BMS-986253	Nivolumab; Nivolumab + Ipilimumab	Melanoma	I/II	281	Active, not recruiting	NCT03400332
		BMS-986253	Nivolumab	Hepatocellular carcinoma Non-small cell lung cancer	II	48	Active, not recruiting	NCT04123379
Promoting differentiation	Nuclear retinoic acid receptors (RAR)/retinoid X receptors (RXR)	All-trans retinoic acid	Pembrolizumab	Metastatic melanoma	I/II	26	Completed	NCT03200847
		All-trans retinoic acid	Ipilimumab	Advanced melanoma	II	10	Completed	NCT02403778
		All-trans retinoic acid	Nivolumab	Pancreatic cancer	I	10	Active, not recruiting	NCT05482451
		All-trans retinoic acid	Retifanlimab	IDH-mutant glioma	II	55	Recruiting	NCT05345002
		All-trans retinoic acid	Tislelizumab + chemotherapy	Esophageal squamous cell carcinoma	II	180	Not yet recruiting	NCT06703047
		All-trans retinoic acid	Toripalimab	Triple-negative breast cancer	–	32	Not yet recruiting	NCT06371274
		All-trans retinoic acid	Cemiplimab	Advanced leiomyosarcoma	II	16	Not yet recruiting	NCT06528769
		All-trans retinoic acid	Atezolizumab	Metastatic lung non-small cell carcinoma Recurrent lung non-small cell carcinoma Stage IV lung cancer AJCC V8 Stage IVA lung cancer AJCC V8 Stage IVB lung cancer AJCC V8	I	18	Recruiting	NCT04919369
		All-trans retinoic acid	Bevacizumab; atezolizumab	Colorectal cancer	II	22	Recruiting	NCT05999812
Inhibiting function	JAK1/2	Golidocitinib	Anti-PD-1	Locally advanced or metastatic non-small cell lung cancer	Ib	30	Not yet recruiting	NCT06690671
		Ivarmacitinib	PD-1/PD-L1 inhibitors	Non-small cell lung cancer	II	86	Not yet recruiting	NCT06925048
		Ruxolitinib	Pembrolizumab	Metastatic malignant neoplasm in the bone Triple-negative breast carcinoma Stage IV breast cancer AJCC v6 and v7	I	12	Completed	NCT03012230
		Ivarmacitinib	Camrelizumab	Unresectable recurrent or metastatic triple-negative breast cancer	II	40	Not yet recruiting	NCT06731153
		Ruxolitinib	Nivolumab	Hodgkin lymphoma	I/II	54	Recruiting	NCT03681561
	STAT3	Napabucasin (BBI608)	Nivolumab	Microsatellite stable, refractory colorectal cancer	II	90	Completed	NCT03647839
		AZD9150	Durvalumab	Advanced solid tumors Relapsed metastatic HNSCC	I/II	340	Active, not recruiting	NCT02499328
Depleting MDSC	MDSC	Gemcitabine/Cisplatin	Toripalimab	Locally advanced head and neck squamous cell carcinoma	I	20	Recruiting	NCT04947241
		Gemcitabine/Cisplatin	Anti-PD-1	Nasopharyngeal carcinoma	III	200	Recruiting	NCT04557020
		Gemcitabine	Pembrolizumab	Biliary tract cancer	II	27	Recruiting	NCT06001658
		Cisplatin						
		Gemcitabine	MK-3475	Previously treated advanced non-small cell lung cancer	I/II	16	Active, not recruiting	NCT02422381
		Low-dose platinum gemcitabine	Cindilimab	Non-small cell lung cancer	IV	60	Recruiting	NCT05312840
		Gemcitabine/Nab-paclitaxel	Spartalizumab + Canakinumab	Metastatic pancreatic ductal adenocarcinoma	I	10	Completed	NCT04581343
		Gemcitabine	Pembrolizumab	HER2-negative advanced breast cancer	II	36	Completed	NCT03025880
		Cisplatin/5-FU/Oxaliplatin/Capecitabine	Pembrolizumab	Stomach neoplasms	III	1579	Completed	NCT03675737
		Oxaliplatin/Capecitabine/Leucovorin/5-FU	Nivolumab; ipilimumab	Gastric cancer Gastroesophageal junction cancer Esophageal adenocarcinoma	III	2031	Completed	NCT02872116

AJCC, American Joint Committee on Cancer; IL-15, interleukin-15; PD-1, programmed cell death 1; 5-FU, fluorouracil

### Inhibition of neutrophil recruitment in conjunction with ICI therapy

Inhibitors targeting CXCR1/2, such as SX-682, AZD5069, and navarixin, have undergone clinical trials to evaluate their efficacy in combination therapies. A recently concluded phase II clinical trial (NCT03473925) assessed the benefit of navarixin (30 or 100 mg) plus pembrolizumab (200 mg) in patients with unresectable stage III or IV castration-resistant prostate cancer (CRPC) (adenocarcinoma), locally advanced unresectable stage III or IV MSS CRC, and stage IV NSCLC lacking effective treatment strategies [141]. Among participants, 4% (2/48) in the 30-mg navarixin cohort and 6% (3/48) in the 100-mg cohort experienced dose-limiting toxicities. Sixty-seven percent of the safety population experienced treatment-related adverse events (TRAEs), with the most prevalent TRAEs being neutrophil reduction, neutropenia, fatigue, and pruritus. No complete responses were observed; 3 patients achieved a partial response, and the median PFS exhibited a dose-independent relationship. Consequently, the study was terminated due to insufficient efficacy. Importantly, a limitation of this trial was the absence of dose adjustments based on real-time monitoring of MDSC activity in peripheral blood or tumor biopsies. Consequently, the dosage could not be promptly escalated when no efficacy was observed, even at high doses of navarixin. Nevertheless, the trial results indicated that the combination of navarixin and pembrolizumab was safe and well-tolerated, without an increased risk of neutropenic fever or infection, although some patients experienced treatment-related neutropenia (10% in the 30-mg group and 17% in the 100-mg group) [141]. This safety profile aligns with previous clinical findings in asthma and chronic obstructive pulmonary disease patients, in whom navarixin was shown to reduce neutrophil counts and improve lung function without elevating infection rates [142,143]. In addition, the limited efficacy in this trial might be linked to the modest decrease in neutrophil counts—few patients had neutrophil counts below 1 × 10^9^/l, which may have been insufficient to overcome neutrophil-mediated immunosuppression and ICI resistance, as the degree of reduction in circulating and TINs correlated with clinical benefit in metastatic CRPC [144]. In addition, the requirement that NSCLC patients had progressed on prior anti-PD-(L)1 therapy may also have influenced outcomes in that cohort. Another CXCR1/2 inhibitor, SX-682, is being evaluated in combination with anti-PD-1 therapy in several phase I/II trials across various solid tumor conditions (NCT04599140, NCT05604560, NCT04477343, NCT05570825, and NCT03161431). Additionally, the safety and efficacy of an immuno-oncology (IO) regimen containing retifanlimab, the triadeno vaccine, the IL-15 agonist N-803, and SX-682 are under investigation in metastatic CRC (NCT06149481). HuMax-IL8 (BMS-986253), a humanized monoclonal antibody against IL-8, has exhibited acceptable safety and tolerability in a phase I clinical trial involving patients with unresectable or metastatic solid tumors (NCT02536469), proposing a scheme for combining IL-8 blockade with other immunotherapies [145]. Of note, although preclinical studies indicate that IL-8 blockade reduces neutrophil chemotaxis, chemotactic changes in neutrophils were not assessed in this phase I study because tumor biopsies were not required [145]. Besides, a number of clinical investigations are projected to assess the benefit of HuMax-IL8 in combination with nivolumab or nivolumab plus ipilimumab in melanoma, NSCLC, and HCC (NCT03400332, NCT04123379). In all, targeting the CXCLs/CXCR1/2 axis seems safe and well-tolerated, while current clinical trials investigating neutrophil-recruitment inhibition combined ICIs remain in early phases (I/II), and clear synergistic benefits are urgently desired to advance their clinical application.

### Neutrophil depletion coupled with ICI therapy

Beyond obstructing neutrophil recruitment, neutrophil depletion can also augment the therapeutic effectiveness of ICIs. Agents like gemcitabine, cisplatin, and 5-FU promote MDSC depletion and are being explored in combination with ICIs. A phase II study (NCT03025880) evaluated the potential synergy of pembrolizumab plus gemcitabine in patients with human epidermal growth factor receptor 2 (HER2)-negative advanced breast cancer; however, the objective response rate (ORR) did not meet statistical expectations, possibly due to the lack of prescreening for PD-L1 expression or tumor-infiltrating lymphocytes (TILs), or because most enrolled patients had received extensive prior therapy. Additionally, no subgroup defined by TIL density or PD-L1 expression exhibited a higher response probability or improved survival [146]. Of note, although this trial achieved limited improvement, patients who experienced therapeutic advantages exhibited a reduction of MDSCs in peripheral blood. This aligns with prior evidence indicating that elevated MDSC levels are associated with poor prognosis [146]. However, the relatively small sample size (*n* = 36) and the heterogeneity of breast cancer subtypes may limit the reliability of these findings. In a completed phase III trial (NCT02872116), first-line nivolumab in conjunction with chemotherapy (capecitabine + oxaliplatin or 5-FU + leucovorin + oxaliplatin) exhibited manageable safety and improved overall survival and PFS compared to chemotherapy alone in advanced gastric cancer, gastroesophageal junction cancer, and esophageal adenocarcinoma [147]. Similarly, another phase III study (NCT03675737) confirmed that pembrolizumab plus chemotherapy (5-FU + cisplatin + capecitabine + oxaliplatin) increased overall survival and PFS with acceptable toxicity over chemotherapy plus placebo in individuals diagnosed with locally advanced or disseminated HER2-negative adenocarcinoma of the stomach or gastroesophageal junction [148]. Therefore, both nivolumab and pembrolizumab, when combined with chemotherapy, demonstrated a manageable safety profile and obviously improved overall survival compared to chemotherapy alone in these trials. This supports their potential as first-line treatment options for the patient population studied. Notably, although the meaningful survival benefits observed in these trials were not directly correlated with reduced neutrophil counts, the combination therapy group did show a decrease in neutrophil counts relative to the placebo group. However, direct evidence linking neutrophil depletion to favorable clinical outcomes remains lacking.

### Advancing neutrophil differentiation in conjunction with ICI therapy

ATRA is frequently observed to reduce MDSCs by promoting their differentiation in oncology patients, and the combination of ATRA with ICIs has been extensively assessed in clinical trials across various cancers. In a phase II clinical study (NCT02403778), treatment with ATRA plus ipilimumab decreased the frequency of circulating MDSCs compared to ipilimumab monotherapy in patients with advanced melanoma and appeared safe without increasing the grade 3 to 4 adverse events [149]. Nevertheless, the cohort size was too small to adequately assess the survival benefits of this combination strategy. Despite this limitation, the preliminary trial results reinforce evidence that ATRA can safely target MDSCs. Another phase Ib/II clinical trial (NCT03200847) combining pembrolizumab with ATRA in metastatic melanoma also showed satisfactory safety and tolerability, accompanied with encouraging response rates [150]. Notably, this combination therapy remarkably reduced the level of circulating PMN-MDSCs, with the reduction being more pronounced in responders [150]. However, several limitations temper the strength of these conclusions. First, this was a single-institution trial with a relatively small sample size. Second, the single-arm design lacked a control group receiving pembrolizumab monotherapy, which precludes a clear assessment of the individual contributions or correlations of each compound to the clinical response. Regardless, targeting PMN-MDSCs through ATRA provides potential for improving ICI responses. However, it should be noted that the aforementioned clinical trials are primarily confined to melanoma. Currently, a growing number of trials are assessing ATRA plus ICIs in other malignancies, such as pancreatic cancer, glioma, breast cancer, and additional tumor types (NCT05482451, NCT05345002, NCT04919369, NCT05999812, NCT06703047, NCT06371274, and NCT06528769). Collectively, the combination of ATRA and ICI represents a promising direction in cancer immunotherapy.

### Reprogramming neutrophil immunosuppression combined with ICI therapy

The immunosuppressive role of TANs represents a major barrier to the efficacy of ICIs, prompting clinicians to investigate targeted agents that can inhibit or reverse this suppression. The JAK/STAT3 signaling pathway mediates neutrophil immunosuppression, positioning JAK and STAT3 as compelling therapeutic targets for modulating neutrophil activity in cancer treatment [118,151]. In a clinical trial (NCT03681561), the JAK inhibitor ruxolitinib combined with nivolumab achieved a disease control rate of 64% and a complete response rate of 31.5% in Hodgkin lymphoma patients unresponsive to ICI therapy, with a median response duration exceeding 1 year [152]. Notably, ruxolitinib treatment substantially reduced both the percentage of circulating PMN-MDSCs and their expression of immunosuppressive genes during ICI therapy. This dual effect provided greater benefit to antitumor immune responses compared to strategies combining neutrophil depletion with ICIs [152]. Likewise, another JAK1 inhibitor itacitinib improved the efficacy of anti-PD-1 therapy through altering the dynamics of T cell differentiation in metastatic NSCLC patients who had progressed on initial anti-PD-1 treatment (NCT03425006) [153]. Unlike the aforementioned clinical trial, which found that JAK inhibition primarily affects PMN-MDSCs, this trial revealed that JAK blockade acts on type I IFN signaling in T cells. One possible explanation for this discrepancy is that the present trial did not focus on the effect of the JAKi on myeloid cells but instead emphasized its T-cell-mediated effects, which may themselves be regulated by PMN-MDSCs. Alternatively, the effector cells of JAKi may vary in a context-dependent manner. Furthermore, inhibition of STAT3—another attractive target in the JAK/STAT pathway—with AZD9150 has been shown to be safe and well-tolerated in patients with diffuse large B-cell lymphoma (NCT01563302). Its efficacy in combination with ICI therapy, however, remains under investigation [154]. Additional studies targeting STAT3 signaling in conjunction with ICIs are ongoing. For instance, an active phase I/II study (NCT02499328) is evaluating durvalumab coupled with either the STAT3 inhibitor AZD9150 or the CXCR2 antagonist AZD5069 in patients with advanced solid tumors or relapsed/metastatic HNSCC. In conclusion, given the intricate regulatory mechanisms of neutrophil immunosuppression, multiple therapeutic targets remain of high clinical interest, and further prospective trials are anticipated.

## Conclusion and Prospects

At present, ICIs represent a pivotal modality in cancer immunotherapy, designed to rejuvenate immune response against tumors and promote immune-mediated elimination of malignant cells via obstructing coinhibitory signaling cascades. Consequently, several ICI drugs have received clinical approval, showing commendable outcomes in certain cancer types. Nevertheless, only a portion of patients respond effectively to ICI therapy due to intrinsic or acquired resistance, or inconsistent efficacy. The high expectations for ICIs, coupled with unresolved challenges of immune resistance, underscore the urgent need for innovative approaches to enhance ICI effectiveness. Immune cells within the tumor microenvironment, particularly those infiltrating into tumors, constitute the cellular foundation of cancer immunotherapies. Emerging evidence indicates that neutrophil frequency and function are obviously associated with ICI efficacy, positioning neutrophils as viable targets for overcoming immune resistance and optimizing ICI outcomes. Thus, a deeper comprehension of neutrophil functions and their regulatory mechanisms in ICI therapy may reveal new therapeutic opportunities.

In clinical trials combining neutrophil-targeted approaches with ICIs, many patients who responded positively exhibited reduced neutrophil counts or diminished immunosuppression in the tumor microenvironment. Incorporating neutrophil-modulating therapies obviously improved the efficacy of ICI. Among these therapies, ATRA not only promotes the maturation of MDSCs but also mitigates their immunosuppressive functions. In a trial of 24 metastatic melanoma patients, ATRA (150 mg/m^2^) in combination with pembrolizumab (200 mg Q3W) achieved an ORR of 71%, with 50% attaining complete remission [150]. Notably, this combination markedly decreased the prevalence of circulating PMN-MDSCs without affecting M-MDSCs [150], underscoring the critical role of PMN-MDSCs in optimizing ICI efficacy. Beyond ATRA, other neutrophil-targeted agents have demonstrated appreciable results. For instance, both HuMax-IL8 (which inhibits neutrophil recruitment) and the JAK inhibitor itacitinib (which modulates neutrophil immunosuppression) have shown safety and tolerability when combined with ICIs. In particular, itacitinib coupled with anti-PD-1 immunotherapy markedly improved response rates in patients with metastatic NSCLC [153]. Therefore, results from ongoing clinical trials in this area are eagerly awaited.

Nonetheless, while there has been substantial advancement in targeting neutrophils alongside ICIs, challenges remain. Given the heterogeneity in the developmental and maturation states of TANs, their transcriptional profiles and roles in modulating the tumor immune microenvironment and responding to ICIs may be inconsistent. Current strategies aimed at depleting neutrophils or inhibiting their recruitment often lack specificity for neutrophil subsets, which may cause analogous interventions to yield disparate outcomes. For example, while CXCR2 inhibition with AZD5069 facilitates immunotherapy in HCC by eradicating PMN-MDSCs [85], the same combination in NASH–HCC promotes neutrophil accumulation and reprogramming toward an antitumor phenotype [88]. This discrepancy may arise from shared characteristics such as surface markers and the multilobed nuclear structure between PMN-MDSCs and neutrophils, as well as differences in cancer types. Hence, employing proteomics and genomics techniques to standardize the nomenclature and characterization of various neutrophil subtypes may be essential. Besides, CXCR2 is broadly expressed on both protumor and antitumor neutrophils and interacts with multiple ligands. Blocking CXCR2 therefore disrupts not only the IL-8/CXCR2 signaling axis but also signaling by other CXCR2 ligands. Thus, selective inhibition of protumor neutrophil subtypes holds substantial promise for improving the therapeutic effectiveness of current ICIs. Given the complexity and heterogeneity of the tumor microenvironment, emerging nanomaterial platforms may enhance targeting precision and therapeutic effects while minimizing systemic toxicity [155–157]. Future efforts should explore nanomaterials that specifically target distinct neutrophil populations. Moreover, in addition to T-cell-focused checkpoints such as PD-1/PD-L1 and CTLA-4, immune checkpoints on NK cells—such as killer-cell immunoglobulin-like receptors and natural killer group 2 member A—also show clinical potential [15]. This underscores the necessity to cotarget multiple immune checkpoints for breakthroughs in cancer therapy.

Many combination approaches are still in the preclinical stage, with limited clinical evaluation and translational relevance. Existing treatment strategies have not adequately met clinical needs, as demonstrated in both preclinical and clinical trial data. For instance, although targeting NETs alongside immune checkpoints shows preclinical promise, no clinical applications have emerged. One reason for its backwardness is partly due to the dual function of NETs under different cancer types; moreover, NETs formed in different locations such as blood vessels, tissues, or tumors may have different functions, and current means in clinics fail to precisely target NETs at specific sites. Hence, understanding the spatial background of NET formation and unraveling its opposite effects in cancer are crucial. Furthermore, only a restricted number of drugs targeting immunosuppression have been authorized for clinical use, and the success rate for experimental drugs remains notably low [158]. Limited efficacy, irreversible side effects, poor stability, and inadequate tumor delivery—e.g., of oligonucleotide-based STAT3 inhibitors—hinder clinical translation [159]. Moreover, the efficacy of inhibitors is contingent upon their concentration and selectivity [160]. Pathways such as the JAK/STAT3 pathway not only regulate the activation of neutrophils but also play crucial roles in multiple physiological processes; indiscriminate blockade may result in systemic side effects. Consequently, more selective inhibitors are needed to improve efficacy and safety. Although preclinical investigations support the synergy of neutrophil-targeted agents with ICIs, much evidence comes from drug-sensitive murine models and cell lines, which may overestimate therapeutic effects compared to human trials, yielding limited clinically applicable data. Moreover, toxicity and irAEs are also needed to be considered in clinical trials. The occurrence of irAEs presents a challenge, with key unresolved issues including the absence of reliable predictive biomarkers for high-risk patients and the difficulty of managing irAEs without compromising antitumor efficacy [161]. Several factors can influence irAE development, such as combination regimens, drug interactions, dosing sequence, tumor type, genetics, and autoimmune status. For instance, dual ICI therapy has been associated with increased irAE frequency and severity, likely due to excessive T cell activation and subsequent tissue damage [162]. Therefore, future research should integrate multiomics (genomics, microbiome, and proteomics) to develop predictive biomarkers and monitoring systems. Given that irAEs and antitumor responses may share underlying mechanisms, elucidating these connections could inform personalized dosing strategies. Furthermore, optimizing drug dosage, frequency, and administration sequence may help achieve a better balance between treatment efficacy and safety.

Due to the variability in response rates and irAE risks, it is urgent to identify efficacious predictive biomarkers to identify patients likely to benefit from specific combination therapies. Current biomarkers such as PD-L1, TMB, and mismatch repair deficiency have clinical utility but exhibit variable predictive performance. Consequently, more candidates, such as TILs and circulating tumor DNA, were emerging, but their predictive value needs to be further validated in clinical practice. Tertiary lymphoid structures (TLSs), unencapsulated ectopic lymphoid organs in nonlymphoid tissues of chronic inflammatory sites, exhibit tremendous potential in predicting antitumor immunity and treatment response. TLSs contain diverse subtypes of B cells, T cells, dendritic cells, and high endothelial venules, and their presence correlates with favorable outcomes [163,164]. A key mechanism involves enhanced infiltration of effector cells and up-regulated genes related to activation (e.g., CD28, CD40/CD40LG, ICOS, CTLA4, CD80, and CD86), migration (e.g., CCR7 and SELL), antitumor polarization (e.g., TBX21, IFNG, and IL2), and cytotoxicity (e.g., GZMB and PRF1) [165,166]. Thus, TLS development may offer new biomarkers for ICI combination therapy. In addition, immunoproteomics, a specialized branch of proteomics, has displayed utility in vaccine development and diagnostic biomarker discovery [167], and may help identify novel neutrophil-related targets. Besides, according to a bibliometric and visualized study, multiomics application, integrating genomics, proteomics, transcriptomics, epigemomisc, metabolomics, microbiomics, and others, provides substantial capability in drug discovery and comprehensive view of disease progression from a molecular perspective [168,169]. Artificial intelligence (AI), especially machine learning, which is one of the most successful applications of AI, can help parse complex omic data and further improve the integration and application of multiomics [170]. Therefore, the conjunction of multiomics with machine learning may uncover new therapeutic targets and biomarkers, advancing cancer therapy toward personalized precision medicine.

Together, extensive research and clinical trials are urgently required to explicate the underlying mechanisms of neutrophil–ICI combinations, identify predictive biomarkers, and optimize therapeutic protocols to improve patient outcomes.
